# Synthesis and Biological Evaluation of Novel Mono Acid Esters Derived from the Constituents of *Urtica pilulifera*

**Published:** 2014

**Authors:** Ahmad I. Husein, Waheed J. Jondi, Nidal A. Zatar, Mohammed S. Ali-Shtayeh

**Affiliations:** a *Biodiversity and Environmental Research Center (BERC), Nablus , Til, Palestine .*; b *Chemistry Department, An- Najah National University , Nablus , Palestine .*

**Keywords:** Hydroxybenzoic acids, 2-phenoxyethanol, Antioxidant, Antimicrobial, Anticancer

## Abstract

New mono acid esters have been synthesized from the reaction of benzoic acid and mono-hydroxybenzoic acids with 2-phenoxyethanol separated from *Urtica pilulifera*, characterized, and screened for possible antioxidant, antifungal, antimicrobial and anticancer activities.

These phenolic acid esters gave various degrees of free radical scavenging, but the values were lower than that of *α*-tocopherol. The concentrations of the tested compounds needed to reduce DPPH absorption by 50% at 517 nm were nearly in the range of 900-1100 µg/mL. While for α-tocopherol was 40 µg /mL. The compounds were tested *in-vitro* against six bacterial species which are known to cause dermic and mucosal infections in human. 2-phenoxyethyl benzoate showed significant activity in the range of 30% against *P. aeruginosa* to 70% against *E. coli* compared with the activity of Streptomycin. On the other hand 2-phenoxyethyl 2-hydroxybenzoate reveals 70% of gentamicin against *K. pneumoniae. *The tested compounds also showed complete inhibition at a concentration less than 37.5 µg/mL against *M. canis* and less than 50 µg/mL against *T. rubrum*. 2-phenoxyethyl 4-hydroxybenzoate showed considerable activity against MCF-7 with IC_50_ is less than 62.5 µg/mL.

## Introduction

Many people believe that medicinal plants are more natural and more accessible than manufactured drugs. In addition to nutritional components, medicinal plants were used in treating a wide spectrum of ailments and diseases, and they have been screened for their potential uses as alternative remedies and the preservation of foods from toxic effects of oxidants (1). A large number of plants have been found to contain ingredients that have antibacterial, antifungal, and anticancer activities. Other plants are used in traditional medicine due to their antioxidant properties (2, 3). *Urtica pilulifera* is commonly known in Roman as Nettle and in Palestine as (Qurraus), has been long used in many countries around the world as a traditional medicine for curing sore joints by mixing the plant juice with oil. The contents of the stinging hair provide a cure for rheumatism, hemorrhage. Decoction of the summits of the plant is diuretic, depurative. Seeds are used for renal stones and inflammation of the bladder, diuretic and aphrodisiac. It is used in the treatment of various diseases including Diabetes Mellitus and other ailments (4-6). Plants have an almost limitless ability to synthesize chemical substances mainly secondary metabolites, of which at least 12000 have been isolated, a number estimated less than 10% of the total (7). Chemical analysis of plant’s extracts showed the existence of many chemical compounds related to different classes such as alkaloids, quinines, sugar alcohols, terpenes, polyphenols, flavonoids, phenolics and many others. Flavonoids and phenolics and their derivatives are found in most plants. Such compounds are very important for both humans and plants (8). They act as cell wall support materials (9) and as colorful attractants for birds and insects helping seed dispersal and pollination (3). Flavonoids and phenolic acids also have antioxidative (10) and anticarcinogenic effects (11). Phenolic acids are large and heterogeneous group of biologically active non-nutrients. They are present in plants as hydroxylated derivatives of benzoic and cinnamic acids (12, 13). Phenolic compounds are important in the defense mechanisms of plants under different environmental stress conditions such as wounding, infection, and excessive light or UV irradiation (14). Phenolics are not only unsavory or poisonous, but also of possible pharmacological value (15). They are two main groups of phenolic compounds, the first is hydroxybenzoic acids which have a general structure derived directly from benzoic acid. The variations in the structures of individual hydroxybenzoic acids lie in the hydroxylations and methylations of the aromatic ring (16). The second is hydroxycinnamic acids usually occur in various conjugated forms, which are esters of hydroxy acids or sugar derivatives (17). 

The aim of this work was to synthesize more active compounds from the constituents of medicinal plants collected from different locations in the northern West Bank of the Palestinian Authority. 

In extension to our work towards the synthesis of biologically active compounds, we investigate their *in-vitro* biological activities.

## Experimental

*General*

Synthesized compounds were purified by chromatography on a silica gel 60 (230-400 mesh; Merck) column and identified by thin-layer chromatography (TLC), UV, IR and NMR. Melting points (mp) were taken on a BUCHI 530 apparatus. TLC was performed on pre-coated silica gel F254 plates (Merck) using a 254-nm UV lamp to visualize the compounds. IR spectra were recorded on a Shimadzu Furrier Transform Infrared FTIR-8700 Spectrophotometer using Nujol as mulling agent; only the most significant absorption bands are reported in cm^-1^. ^1^H and ^13^C NMR spectra were recorded at room temperature using a 300 MHz Bruker DPX spectrometer. Chemical shifts were recorded in parts per million (δ) in CDCl_3_ with tetramethylsilane (TMS) as internal reference. A Perkin–Elmer Lambda 40 UV/VIS spectrophotometer was used in the 1, 1-diphenly-2-picrylhydrazyl-hydrate (DPPH) scavenging and β-carotene assays. 

*Chemicals and materials*

β-carotene, linoleic acid, (DPPH) and α-tocopherol were purchased from Sigma,(Sigma, Aldrich GmbH, Sternheim, Germany). While Tween-40, Folin-ciocalteu’s phenol reagent (FCR), sodium carbonate, ethanol, chloroform and other chemicals and reagents were purchased from Merck (Darmstat, Germany). Trypsin, RPMI 1640 culture medium, fatal calf serum, glutamine, amphotricine B, Hank’s balanced solution, Trypan blue solution, penicillin and gentamicin, all other reagents are of analytical grade.

*General* *s* *ynthetic procedure for acid ester and mono-hydroxy acid ester*

Acid esters were synthesized by refluxing benzoic acid or mono-hydroxybenzoic acids with 2-phenoxyethanol separated from *Urtica pilulifera* (18) for five hours. The reaction was left at room temperature for 24 h. The product was dried from the solvent, and the residue was partitioned between ethyl acetate and saturated NaHCO_3_. The organic phase was washed with brine, dried over Na_2_SO_4_, and the solvent was evaporated. The residue was purified by flash chromatography on a silica gel column. Yields are between 62–83%. 

**Figure 1:**
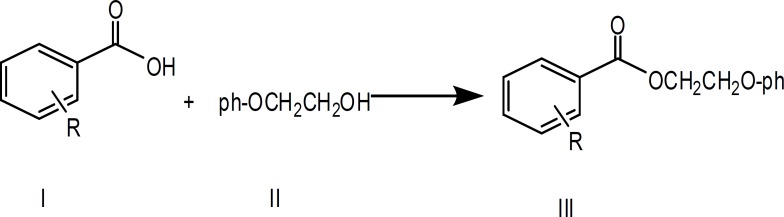
Preparation of 2-phenoxyethyl benzoate (III, R = H)

Benzoic acid (10.0g, 0.08 mol) was reacted with 2-phenoxyethanol (II) (11.0g, 0.08 mol) and the final product was purified by flash chromatography using (n-hexane /ethyl acetate 3:1) as eluent (mp 112-114 ºC); yield 62%. UV λmax 240-270. IR *ν*_max_ (1697, 1596, 1242, 1064 cm^-1^). 

^1^H-NMR (CDCl_3_); δ : 8.1 (2H, m, H2 and H6); 7.55 (1H, m, H4); 7.33 (2H, m, H3 and H5); 7.30 (1H, m, H4); 7.20 (2H, m, H3 and H5); 6.90 (2H, m, H2 and H6); 4.45 ( 2H, t, *J*= 6Hz, CH_2_ ); 4.31 (2H, t, *J*=6 Hz, CH_2_) ppm. 

^13^C-NMR (CDCl_3_) δ : 177,C-ester; 158, C1’; 132.9, C-4; 130.5 C-1; 130.1, C-3’ and C-5’ ; 129.0, C2- C6; 128.0, C3; 121.0 C4’; 114.0 ,C2’-C6’; 69.4, CH_2_; 66.9 , CH_2_ ppm.

*Preparation of 2-phenoxyethyl 2-hydroxybenzoate (III, R = 2-OH)*

2-Hydroxybenzoic acid (10.0 g, 0.07 mol) was reacted with 2-phenoxyethanol (11.0 g, 0.08 mol) and the product was purified by flash chromatography using (n-hexane /ethyl acetate 3:7) as eluent (mp 104-106 ºC); Yield 71%. UV λmax 240-270 . IR (3309, 1712, 1596, 1242, 1072 cm^-1^) . ^1^H-NMR (CDCl_3_); δ : 9.70 (1H, s, br ,OH); 7.90 (1H, m, H-6); 7.60 (1H, m, H-4); 7.40 (2H, m, H-3’ and H-5’); 7.10 (2H, m, H-3 and H-4’); 7.00 (1H, m, H-5); 6.9 (2H,m, H-2’ and H-6’); 4.41 ( 2H, t, *J*= 6Hz, CH_2_ ); 4.30 (2H, t, *J*=6 Hz, CH_2_) ppm. 

^13^C-NMR (CDCl_3_) δ : 172,C-ester; 166, C-1’; 156, C-2; 135, C-4; 131, C-6’;130 (C-3’ and C-5’) ; 121.0, C-5’; 120, C-4’; 119.0, C2’and C-6’; 117.0, C-1; 114.0 ,C3’; 69.2, O-CH_2_; 62.1, CH_2_-O ppm. 

*Preparation of 2-phenoxyethyl 3-hydroxybenzoate (III, R = 3-OH)*

Methyl 3-hydroxybenzoate (10.0 g, 0.07 mol) was boiled with 5 mL 6 M HCl for 10 minutes. The product was mixed with 2-phenoxyethanol (11.0 g, 0.08 mol) and reflux for 3 hours. The reaction was cooled and left at room temperature for 24 hours. The product was collected as solid crystals. The crystals were purified by flash chromatography using (*n*-hexane /ethyl acetate 3:7) as shown in Scheme 3. (mp 138-141^ ͦ^C); Yield 63%. UV λmax 240-270. IR *ν*_max_ (3309, 1689, 1596, 1496, 1242, 1087, 673,694 cm^-1^). ^1^H-NMR (CDCl3); δ 7.45 (2H, m, H-2 and H-6); 7.28 (1H, H-5); 7.04 (3H, m, H-2’, H-6’ and H4); 6.88 (3H, H3’,H5’ and O-H); 6.80 (1H, H4’); 4.44 (2H, t, J=7.0 Hz ,-OCH2); 4.16(2H, t, J=7 Hz, CH2-O) ppm. ^13^C-NMR (CDCl3) δ : 165.3,C-ester; 157.8, C-1’; 156.5 , C-3 ; 132.0, C-1; 129.4, C’-3 and C-5’;125.9, C-5; 124.9, C-6 ; 120.8, C-4’; 117.4, C-2 and C-4; 66.3, O-CH2; 64.5, CH2-O ppm 

*Preparation of 2-phenoxyethyl 4-hydroxybenzoate (III, R = 4-OH)*

4-Hydroxybenzoic acid (10.0 g, 0.07 mol) was mixed with 2-phenoxyethanol (11.0 g 0.08 mol). The product was collected as solid and was purified by flash chromatography using (*n*-hexane /ethyl acetate 3:7) as eluent. (mp 146-148 ºC); Yield 83%. UV λmax 240-270. IR *ν*_max_ ( 3309, 1689, 1596, 1496, 1242, 1087, 694, 673, 484 cm^-1^). ^1^H-NMR (CDCl_3_); δ 7.95 (2H, m, H-2 and H-6); 7.25 (2H, m, H-3 and H-5); 6.95 (3H, m, H-3’, H-6’ and H4’); 4.41 (2H, t, J=7.0 Hz ,-OCH_2_); 4.20 (2H, t, *J*=7 Hz, CH_2_-O) ppm. .^13^C-NMR (CDCl_3_) δ : 166.8,C-ester; 160.4, C-4; 156.8 , C-1’ ; 132, C-2 and C6; 129.9, C-3 and C5’;122.3, (C-3’ and C-5 ) ; 121.0, C-4 ; 115.3, C-1’; 114.3, C2 and C-6’; 69.2, O-CH_2_; 62.2, CH_2_-O ppm. 

*Antioxidant activity*

*DPPH assay*

The hydrogen atom or electron donation abilities of the corresponding compounds were measured from the bleaching of the purple-colored methanolic solution of DPPH. This spectrophotometric assay uses the stable radical DPPH as a reagent (19, 20). One mL of various concentrations of the extracts in ethanol was added to 4 mL of 0.004% methanol solution of DPPH. After 30 minutes, incubation period at room temperature, the absorbance was read against a blank at 517 nm. The percent Inhibition I (%) of free radical by DPPH was calculated as follows:

Equation (1) I (%)=((Ablank– Asample)/ Ablank) x 100

Where A_blank_ is the absorbance of the control reaction (containing all reagents except the test compound), and A_sample_ is the absorbance of the test compound. Extract concentrations providing 50% inhibition (IC_50_) are calculated from the plot of inhibition (%) against extract concentration. Tests were carried out in triplicates.

*β-carotene- linoleic acid*

The antioxidant activity of the synthesized compounds, based on coupled oxidation of β-carotene and Linoleic acid emulsion was evaluated following a modified method of Gazzani and Miller (21). The mechanism of bleaching of β-Carotene is a free-radical-mediated phenomenon resulting from the hydro- peroxides formed from linoleic acid (22). When linoleic acid is incubated at 50 ˚C free radicals are produced. The free radicals will attack the highly unsaturated β-carotene molecule. During the oxidation process β-carotene loses its chromophore and orange color characteristic. The presence of antioxidants can hinder the extent of β-carotene bleaching. Antioxidants are able to slow down the rate of bleaching by neutralizing the linoleate free radicals in the system. Briefly, 1mg of β-carotene was dissolved in 2 mL chloroform and 20 mg of linoleic acid, 200 mg of Tween 40 were added. Chloroform was completely evaporated using a rotary evaporator under reduced pressure at low temperature (less than 30 °C), and 200 mL of distilled water saturated with oxygen were added to the flask with vigorous shaking for 30 minutes. Aliquots (5 mL) of the prepared emulsion were transferred to a series of tubes each containing 0.1 mL of extract or tocopherol (2 mg/mL). A control sample was prepared exactly as before but without adding antioxidants. Each type of sample was prepared in triplicate. The test systems were placed in a water bath at 50 °C for 2 hours. The absorbance of each sample was read spectrophotometrically at 470 nm, immediately after sample preparation and at 15-min intervals until the end (t = 120 min) of the experiment. Antioxidant activities in β-carotene-linoleic acid model were measured by the changes in the absorbance at 470 nm.

*Antibacterial activity testing*

The antibacterial activity of the synthesized compounds was determined against the following microorganisms: *Staphylococcus aureus* (ATCC 25923), *Escherichia coli* (ATCC 25922, and JM109), *Klebsiella pneumoniae* (ATCC 13883), *Proteus vulgaris* (ATCC 13315), and *Pseudomonas aeruginosa* (ATCC 27853), *Trichophyton rubrum*, and* Microsporum canis*. All of the isolates were purchased from BERC /Til Village. Solutions of each of the synthetic compounds (1.0 mg/mL) in DMSO were sterilized by filtration through a 0.45 mm membrane filter*. *Antibacterial tests were then carried out by the disc diffusion method (Yaghmour, 1998) (4) using an inoculum containing 10^6^ bacterial cells / mL spread on Muller–Hinton agar plates (1 mL inoculum/plate). The discs (6 mm in diameter) were soaked with 1.0 mL of the test solution (0.2 mg/disc), placed on the inoculated agar, and incubated at 37 °C for 24 h. All tests were performed in triplicates.

*Antifungal activity testing*

The synthesized compounds were tested for their antifungal activity against the test pathogens using a modified poisoned food technique. Each compound was mixed with the pre-sterilized SDA medium to a concentration of 2.5 mg/mL. A mycelial agar disk of 5 mm diameter was cut out of 12 days old culture of the test fungus and inoculated on to the freshly prepared agar plates. In controls, sterile distilled water was used in place of the test sample. The inoculated plates were incubated in the dark at 24 ˚C and the observations were recorded after 10 days. Percentage of mycelial inhibition was calculated using the following formula:

Equation (2) % mycelial inhibition =(dc-ds /dc) x100%

Where dc is colony diameter of the control, and ds is colony diameter of the sample. All tests were performed in triplicates.

*Anticancer activity testing*

The cells of Breast cancer ( MCF-7 human carcinoma) were cultured in RPMI 1640 medium supplement with 10% heated fetal bovine serum, 1% of 2 mM l-glutamine, 50 IU/mL penicillin, 50 µg/mL amphotricine B. After checking for the absence of mycoplasms and bacteria, cell grown at 35 °C as monolayer confluent cells in RPMI 1640 medium supplemented with 10% calf serum. To avoid cell membrane sensitization, no antibiotics were used. For the assay, cells were washed three times with phosphate buffer saline (PBS). PBS was decanted, cells detached with 0.025% Trypsin – EDTA and RPMI 1640 medium was added to make up a volume of 10 mL. The cell suspension was centrifuged at 1000xg for 10 minutes and the pellet was re-suspended in 10 mL medium to make a single cell suspension. Viability of cells was determined by Trypan blue exclusion and it exceeds 96% as counted in a haemocytometer. Stock cultures were duplicate weekly after inoculation. The cell line was cultured in 6-well tissue culture plates (9.8 cm^2^) and incubated at 35 °C in a humidified atmosphere containing 5% CO_2_. After 24 hours the cells were treated with the compounds. 0.1 mL of each pure compound was diluted to a serial dilutions (500, 250, 125, 62.5 µg/mL).

## Results and Discussion

*Antioxidants*

*DPPH Assay*

Ethanolic solutions (10 mg/mL) of each compound were prepared to study their antioxidant activities. DPPH is one of the methods used to evaluate the antioxidative activity of antioxidants. The free radical DPPH is deep violet in color and posses a characteristic absorption at 520 nm. The deep violet color of DPPH gradually changes to pale yellow (reduced form) when DPPH is exposed to free radical scavengers. The decrease in absorbance at 520 nm induced by antioxidants determines the reduction capability on the DPPH radicals. The higher IC_50_ value indicates lower scavenging activity as more amount of the scavengers were required to achieve 50% scavenging reaction and thus the scavengers are less effective in scavenging the DPPH. As shown in Table 1. These phenolic acid esters gave various degrees of free radical scavenging, but the values were lower than that of α-tocopherol. The concentrations of the tested compounds needed to reduce DPPH absorption by 50% at 517 nm were nearly in the range of 900-1100 µg/mL Table 2. While for α-tocopherol was 40 µg /mL. On the other hand the results show that there is a difference in the antioxidant activity between phenolic acid esters themselves. 

It has been shown that IC_50_ values for 2-phenoxyethyl benzoate and 2-phenoxyethyl 3-hydroxy benzoate are less than 2-phenoxyethyl 2-hydroxy benzoate and 2-phenoxyethyl 4-hydroxy benzoate. IC_50 _values of the phenolic compounds are slightly affected by the molecular structure and the position of hydroxyl group on the site of ortho, meta and para on the benzoic moiety. This is not 

 consistent with studies done on polyphenolic compounds and showed that the structure is not required for the antioxidant activity (23, 24). 

**Table 1:** Percent inhibition of phenolic acid esters at different concentrations

No.	Concentration µg/mL	% inhibition					
		100	200	400	600	800	1000
1	α- Tocopherol	86± 1.23	91± 0.85	92± 1.34	93± 1.65	95± 0.94	97± 1.45
2	2-phenoxyethyl benzoate	17± 0.94	22± 1.48	39± 1.96	47± 1.88	51± 0.78	56± 2.06
3	2-phenoxyethyl 2-hydroxy benzoate	8± 1.19	13± 1.16	19± 1.59	33± 1.42	40± 1.12	46± 2.35
4	2-phenoxyethyl 3-hydroxy benzoate	11± 1.77	21± 0.98	24± 2.03	40± 0.95	46± 1.58	54± 1.85
5	2-phenoxyethyl 4-hydroxy benzoate	13± 1.67	20± 1.52	23± 1.75	31± 1.23	36± 1.63	41± 1.79

**Table 2:** IC_50_ (µg/mL) values of phenolic acid esters

1	α- Tocopherol	˂ 100
2	2-phenoxyethyl benzoate	790 ± 5.48
3	2-phenoxyethyl 2-hydroxy benzoate	˃ 1000
4	2-phenoxyethyl 3-hydroxy benzoate	900 ± 3.68
5	2-phenoxyethyl 4-hydroxy benzoate	˃ 1000

IC_50_ (µg/ml) values expressed are means ± standard deviation of triplicate measurements

*β-carotene-linoleic acid test*

**Figure 2:**
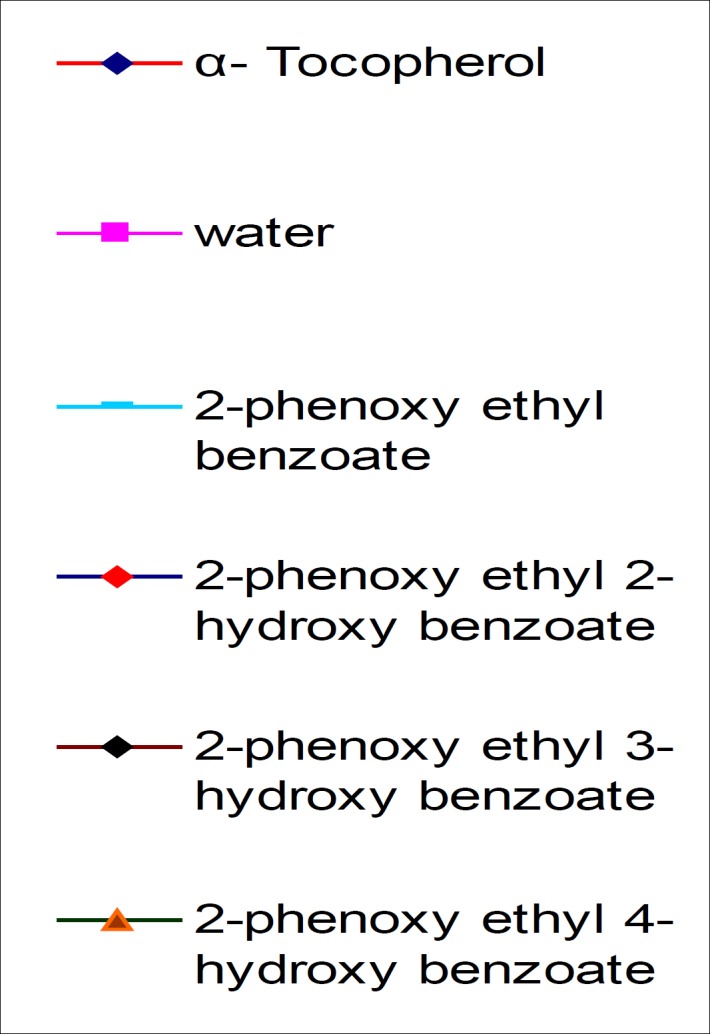
antioxidant activities of modified compounds in β-Carotene-linoleic acid test

The same compounds were tested for their antioxidant activity using emulsion system of β-carotene linoleic acid depending on the fact that β-carotene loses its color in the absence of antioxidants (25, 26).All of the synthetic compounds showed higher antioxidant efficiency compared with water (control) and the synthetic antioxidant α-tocopherol which gave the highest β-carotene degradation (*i.e.* least antioxidant efficiency). Figure 2 showed the antioxidant activities of the synthetic acid esters and positive reference standard with the coupled oxidation of β-carotene. The antioxidant activity of all the compounds gradually increases with the increase of the compound concentration. Water showed the highest β-carotene bleaching activity followed by α-tocopherol after 1 hour. 2-phenoxyethyl benzoate revealed the best antioxidant with absorbance 0.4 after 1 hour compared with the other synthetic compounds. The second one is 2-phenoxyethyl 3-hydroxy benzoate which showed absorbance 0.3 after 1 hour.

*Antibacterial activity*

The entire compounds (Table 3) were tested *in-vitro* against six bacterial species which are known to cause dermic and micosal infections (27) beside other infections in human. All compounds studied in this work showed antibacterial activity. 2-phenoxyethyl benzoate showed significant activity. It revealed in the range of 30% against *P. aeruginosa to* 70% against *E. coli. JM109* compared with the activity of Streptomycin. It is noteworthy to take attention for those modified compounds although they possess less activity than synthetic antibiotics. It is important to prepare new therapeutics because some kinds of bacteria become resistant to certain drugs after a period of time. 

**Table 3:** Antibacterial activity of the compounds

**No.**	**Compound**	**Inhibition zone diameter (mm)** a **Micro-organisms**					
		***P .vulgaris***	***E . coli. JM109***	***E. coli ***	***P. aeruginosa ***	***S. aureus ***	***K. pneumoniae***
S	Streptomycin	14±0.71*	17±0.7	26.3±0.8	30.8±0.8	29±1.2	37.8±1.1
1	2-phenoxyethyl benzoate	6.5±0.5	7±0.7	7±0.7	6.0	6.0	10.8±0.8
2	2-phenoxyethyl 2-hydroxy benzoate	7.8±0.4	11.5±0.9	8.5±0.5	10±0.8	10.8±0.8	23.3±0.8
3	2-phenoxyethyl 3-hydroxy benzoate	6.0	11.5±0.9	6.0	9.5±0.5	6.0	6.0
4	2-phenoxyethyl 4-hydroxy benzoate	6.0	7.0±0.0	8.5±1.1	8.0±0.7	7.5±0.5	8.0±0.0

a ^:^ Disc diameter, 6mm

* inhibition zone diameter in mm ± S.D

*Antifungal activity*

**Figure 3:**
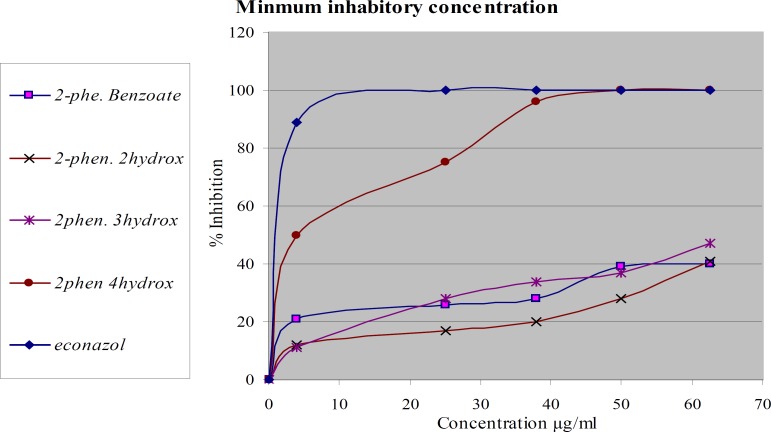
Antifungal activities of modified compounds on* T. rubrum*

**Figure 4:**
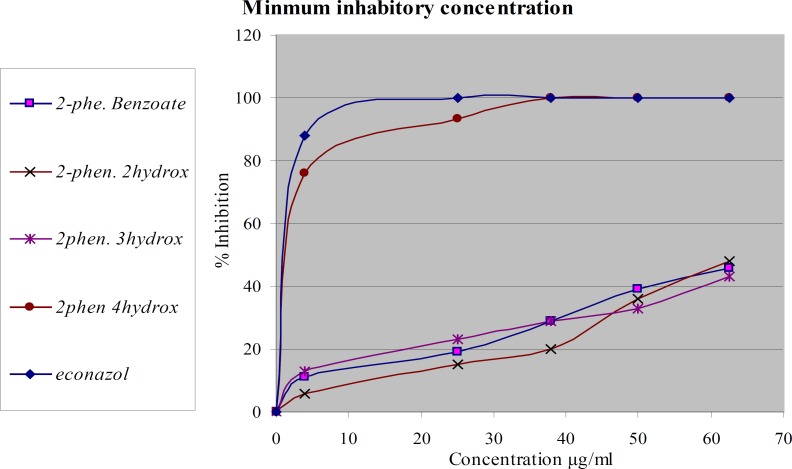
Antifungal activity of modified compounds on* M. canis*

The same compounds were tested again *in-vitro* for their antifungal activity against two types of dermatophytes *M. canis* and *T. rubrum* Figures 3 and 4. The most active compound is 2-phenoxyethyl 4-hydroxy benzoate. It showed complete inhibition at a concentration less than 37.5 µg/mL against *M. canis *and less than 50 µg/mL against *T. rubrum.* The activity of this compound is about 95% of econazole. The other compounds didn’t show significant activity. The tested compounds have the same molecular weights and formulas, but different in structural formula especially in the position of hydroxyl group. These results emphasize the importance of functional group position in the activity of the compound against dermatophytes. 

*In-vitro cytotoxicity*

The results of preliminary cytotoxicity screening of the compounds against breast cancer MCF-7 were summarized in Table 4. 2-Phenoxyethyl 4-hydroxy benzoate showed considerable activity against MCF-7. It showed 36 % viability at 62.5 µg/mL and 11% at 500 µg/mL, while 2-phenoxyethyl 2-hydroxybenzoate showed 52% at 500 µg/mL. This means that IC_50 _for 2-Phenoxyethyl 4-hydroxy benzoate is less than 62.5 µg/mL. these results may be attributed to the structure of 2-Phenoxyethyl 4-hydroxy benzoate by forming inter H-bonds with DNA while 2-phenoxyethyl 2-hydroxybenzoate less active due to formation of intra H-bonds with DNA. 

**Table 4:** percent viability of cancer cells

No	Compound	Concentration µg/mL				
		62.5	125	250	500	1000
1	2-phenoxyethyl benzoate	-	-	-	-	-
2	2-phenoxyethyl 2-hydroxybenzoate	94*	89	72	52	-
3	2-phenoxyethyl 3-hydroxybenzoate	-	-	-	-	-
4	2-phenoxyethyl 4-hydroxybenzoate	36	21	14	11	-

* Percent viability of cell at different concentrations

## Conclusion

In the present work new mono acid esters have been synthesized from the reaction of benzoic acid and mono hydroxybenzoic acids with 2-phenoxyethanol separated from *Urtica pilulifera*. Some of the synthesized compounds exhibited promising antifungal and anticancer activities.

The most active products were 2-phenoxyethyl 2-hydroxybenzoate and 2-phenoxyethyl 4-hydroxybenzoate, with the latter showing remarkable antifungal and anticancer activities. Such activity may be attributed to the molecular structure, and the position of hydroxyl group. Thus, it is worthy to be attention to those biologically active synthesized phenolic acid esters since they are prepared from the constituents of selected medicinal plant as starting materials.
